# Impact of aortic root geometry and degree of aortic calcification on outcomes of patients undergoing TAVI procedure

**DOI:** 10.1177/02676591241248537

**Published:** 2024-05-02

**Authors:** Ramiz Emini, Christopher Gaisendrees, Marie Kreft, Andreas Liebold, Robert Bauernschmitt, Julia Merkle-Storms

**Affiliations:** 1 Department of Cardiothoracic Surgery, Heart Center, 27182 University of Cologne, Germany; 2 Department of Cardiothoracic and Vascular Surgery, 27197 University of Ulm, Germany; 3 Department of Cardiac Surgery, 27243 University Hospital Zurich, Switzerland

**Keywords:** TAVI procedure, aortic leaflet geometry, degree of aortic calcification, paravalvular leak, outcomes

## Abstract

**Aims:**

Adequate differentiation of calcifications in contrast-enhanced CT scans remains difficult to assess TAVI parameters. The size of the aortic leaflets has not been taken into account so far in present studies. The aim of our study was to establish a new method for optimized quantification of the aortic valve calcification degree in contrast-enhanced CT scans for better preoperative prediction of postoperative paravalvular leak after TAVI.

**Methods and results:**

We retrospectively analyzed preoperative contrast-enhanced CT scans of patients who underwent TAVI in our institution between 2014 and 2017. Calcium volume was quantified by a method using contrast enhanced computer tomography (3mensio-Structural Heart-7.2 software) with different iodine contents for better discrimination of contrast agent from calcium and by an individually set Houndsfield Unit (HU) threshold with 50HU above the individually determined reference value. Calcium volume was correlated with surface area of each aortic cusp. Perioperative variables were analyzed. All patients (*n* = 150) with severe aortic stenosis were treated with TAVI implantation. Overall incidence of postoperative trace to moderate PVL was 37%. The amount of calcium correlated with the incidence of PVL. In a logistic regression analysis total volume of calcification (*p* = .032) as well as calcification of each aortic cusp (NC_*p* = .001; RC_*p* < .001; LC_*p* = .001) were independent predictors.

**Conclusions:**

Calcification degree as well as its correlation with the surface area of each aortic cusp significantly influence incidence of PVL. Our new method improves preoperative quantification of the calcification degree by use of contrast agents with different iodine contents and thereby helps to improve patients’ outcomes.

## Key question

By optimizing the method for preoperative calcium quantification incidence of postoperative paravalvular leak can be reduced.

## Key findings

Our optimized method for quantification of the aortic calcification degree specifies preoperative diagnostics and thus predicts postoperative incidence of paravalvular leak in TAVI and thereby improves patient and valve selection to stratify therapy options. Calculation of the exact calcification degree and the aortic geometry in contrast-enhanced CT scans are possible and essential.

## Take-home message

Our precise method for calculating the calcification degree of the aortic root considering also different contrast agents improves risk stratification and thereby reduces incidence of paravalvular leak after TAVI procedure as one of the most severe postoperative complication.

## Introduction

Calcium in the aortic valve complex is known to be predictive of the severe complication of a paravalvular leak (PVL) after transcatheter aortic valve implantation (TAVI).^1,2^ It is assumed that complete valve expansion is hampered by the existence of calcium depending on its degree.^1,3^ Postoperative incidence of PVL significantly and negatively influences early and long-term outcomes after TAVI implantation.^3,4^ Unfortunately, a structured standardization for assessing the aortic calcification degree is still missing in contrast to the coronary artery calcium assessment once using the Agatston score.^5,6^ This heterogeneity has led to a limited understanding of relevant volumes of calcium influencing development of a postoperative PVL. Although it has been appreciated that cross-sectional measurements of the aortic annulus using contrast-enhanced computer tomography offer the most accurate dimensions for TAVI sizing, the interaction of the aortic calcification degree and distribution with precisely measured annular dimensions remains poorly understood.^5^ Falsification due to contrast medium may lead to over or underestimating of the calcification degree depending on the set Hounsfield unit threshold.

Using contrast agents with different iodine contents and incorporating also the surface area and calcium quantification of each aortic cusp, we sought to improve already existing methods for calcium quantification without falsification due to contrast medium. Especially using computer tomography, the selection of the appropriate calcium threshold is elementary in order to avoid falsification due to contrast medium. Improved estimation of aortic calcification degree offers possibility to reduce incidence of postoperative PVL and might lead to a better standardization of preoperative diagnostics.

## Methods

### Study population

We retrospectively analysed 150 patients receiving a TAVI implantation between 2014 and 2017. All patients who underwent TAVI for symptomatic severe stenosis of the native aortic valve were included in the study. Severe aortic stenosis was defined following international guidelines.^7^ Patients were equally either treated with the ballon-expandable Edward Sapien 3, Direct Flow Medical or the self-expanding CoreValve Evolut performed under predominantly fluoroscopic guidance. The results have been always the same independently from the type of prothesis. In order to correct reliably for precise annular sizing only patients with available contrast CT data for annular dimension assessment were included in this study. In our study all patients have been predilated. Postdilation was performed in seven cases resulting in PVL < Trace in all cases. Postdilation was conducted with a recommended ballon from the manufacturer or a ballon that was not bigger than the minimun annulus diameter +2 mm. Exclusion criteria were bicuspid aortic valve, pure aortic regurgitation and aborted procedures because of annulus >30 mm. Clinical and operative data were prospectively collected in our institutional database. The study complies with the Declaration of Helsinki a locally appointed Ethics Committee approved the research protocol.

### Procedure indication by the heart team

According to the international guidelines, indication for TAVI was discussed within a Heart team including cardiologists and heart surgeons evaluating together patients with severe aortic valve stenosis. All patients with frailty factors who were judged as inoperable or at too high risk for surgery were considered for TAVI.

### Computed tomography and calcium quantification

**Figure 1.**
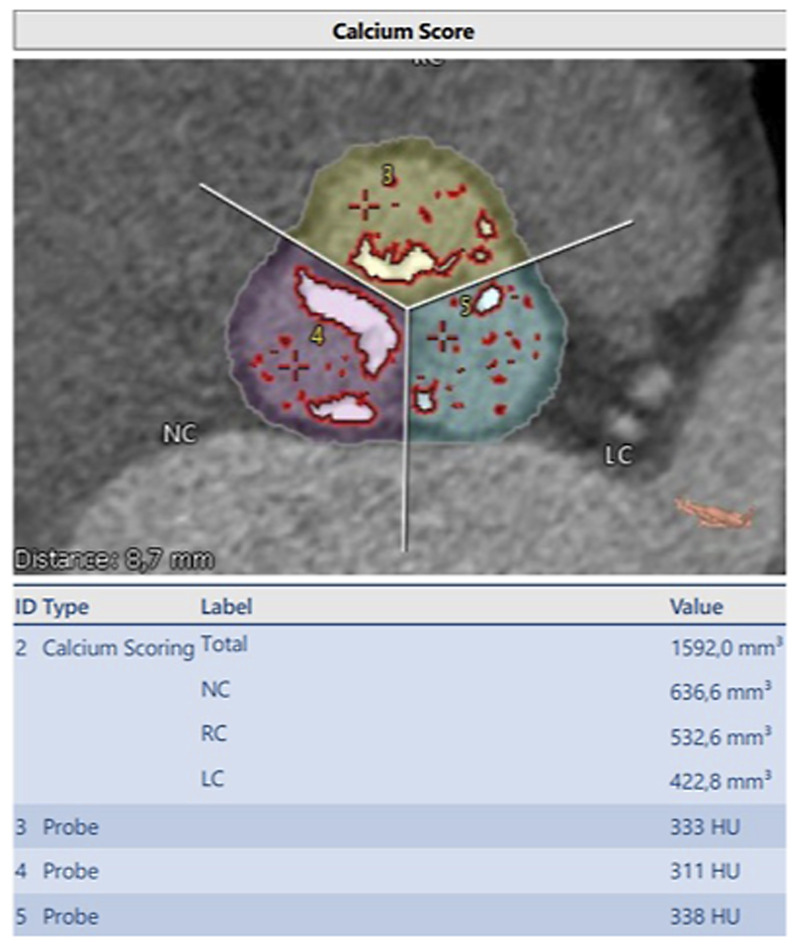
Calcium volume of each cusp (LC = Left coronary cusp, RC = Right coronary cusp, NC = Noncoronary cusp and Total) adjusted to the individualized cut-off value of (probe 1 + probe 2 + probe 3)/3 = 327.33 + 50 HU = 377 HU cut-off value.

All patients underwent 3-dimentional contrast-enhanced ECG-gated multidetector computed tomography for assessment of aortic root anatomy. In our center, all CT scans were analyzed by our team using 3mensio Structural Heart 7.2 software. Aortic geometry and calcium volume in the aortic valve were retrospectively measured using a special software tool called “aortic root workflow assistant”. Our software automatically performs aortic valve cusp delineation and detects all calcified areas for the selected Hounsfield unit (HU) threshold at each of the three cusps. 3mension used standard Hounsfield threshold of 450 HU. In our study we used contrast agents with different iodine contents. In order to taking this into account, we calculated an individual calcium threshold for each patient. For this we calculated a reference value for not calcium containing structures using three voxel probes. These voxel probes were calculated by: Three points in volume of interest were set in the area of lowest opacity and the resulting Hounsfield unit was determined. Mean value of the three points represents the reference value of the patient for non-calcium tissue. Our calcium threshold was thus set 50 HU above the individually calculated reference value as shown in Figure 1. Thus, all voxel with CT values above the reference valve were identified as calcium. Our software quantified calcium volume for each aortic cusp. This new method offers maximal sensibility for calcium detection and avoids false identification of contrast agents as calcium. Measurement of calcium volume was performed by a cardiac surgeon experienced in TAVI and trained in the use of 3mensio software. Interobserver variability was tested in a sampling rate.

### Calculation of the aortic valve geometry

For calculation of aortic valve geometry determination of the aortic annulus is elementary. A centerline through the left ventricular outflow tract, aortic valve and the ascending aorta is necessary and is constructed by the software 3mensio automatically. This line can manually be optimized. In a next step the lowest point of each aortic cusp is determined. Based on these parameters the software constructs the annulus plane. Furthermore, we measured the area of each cusp separately to create an index of Calcium amount per area.

### PVL quantification

PVL was quantified by intraoperative aortography and transthoracic echocardiography (TTE) performed by a dedicated cardiologist. To obtain maximum accuracy, a multiwindow and multiparametric approach was applied. The PVL was categorized none, mild, moderate and severe according to VARC-2 criteria.^8^

### TAVI procedure

All procedures were performed in a hybrid operating room under fluoroscopic control, general anesthesia or conscious sedation, periprocedural TTE and a cardiac perfusionist with ready-to-use cardiopulmonary bypass on site. All implants were performed by a multidisciplinary team. Selection of prosthesis type is agreed preoperatively with the cardiologist and cardiac surgeon on the basis of several parameters including need for elective PCI after TAVI, annulus dimension, and distance from the aortic annulus to the coronary ostia.

### Statistical analysis

The Statistical analysis was performed independently by the first author and the statistician using SPSS software for windows (IBM SPSS Statistics, Release 21.0.0, SPSSInc., Chicago, IL, USA). Data consistency was analyzed for outliers and normality with QQ plots. Additionally, continuous variables were also tested for normality by using Kolmogrov-Smirnov test. Continuous variables were analyzed by two-tailed Student’s *t* test while for non-normally distributed variables by the Mann–Whitney U-test. Univariate and multivariate logistic analysis were used to assess the association for the indexed Calcium/Area Value and the occurrence of paravalvular leak. All reported tests were 2-sided, and *p* values <0.05 were considered as statistically significant.

Continuous variables are displayed as mean ± standard deviation and median values (first and third interquartile range) for non-normally distributed variables. Categorical data are given as frequencies and percentage. A receiver operating characteristic (ROC) curve was calculated, allowing prediction of PVL. Clinical, procedural, ECG, and preoperative MDCT variables were entered into univariate analysis.

## Results

Between 2014 and 2017, a total of 150 patients received a TAVI at the authors’ institution. Patient clinical and procedural characteristics are shown in Table 1. Patients with bicuspid aortic valves were excluded. The mean age was 80 (±5.7) years and 57.3% of all patients were males. Median EuroSCORE II was 5.1 indicating a medium surgery risk (Table 1). Mean aortic annulus diameter was 24.8 (±2.2) mm. Mean aortic area was 484.7 (±86.0) mm^2^. The aortic cusp with heaviest calcification was the non-coronary cusp with 628.6 (356.1; 1246.6) mm^3^ calcium volume, whereas the right coronary cusp showed 573.8 (274.6; 1222.1) mm^3^ and the left coronary cusp 532.8 (260; 1014.5)) mm^3^ (Table 1). Most patients showed a ventricular ejection fraction of almost 60%.

**Table 1.** Baseline characteristics and MDCT measurements.

Characteristics	Values are *n* (%), *n* (median Q1;Q3) or *n* (mean ± SD) in relation to total *n* = 150
Age (years)	80.0 (±5.7)
Female gender	64 (42.7%)
BMI (kg/cm^2^)	27.2 (±4.4)
EuroSCORE II (%)	5.13 (3.01; 9.71)
STS-score (%)	5.3 (3.63; 7.53)
Comorbidities	
Hypertension	145 (96.7%)
Hypercholesterolemia	115 (76.7%)
Diabetes mellitus	50 (33.3%)
Chronic lung disease	34 (22.7%)
Chronic kidney disease on dialysis	104 (69.3%)
	1 (0.6%)
Pulmonary hypertension	43 (28.7%)
Peripheral artery disease	93 (62%)
Coronary artery disease	94 (62.7%)
Mitral regurgitation ≥ mild	68 (45.3%)
Effective aortic valve orifice area (cm²)	0.73 (±0.16)
∆Pmax (in mmHg)	66.2 (±22.7)
Left ventricular function (%)	59.9 (±14.3)
Minimum diameter annulus (mm)	21.8 (±2.4)
Maximum diameter annulus (mm)	27.8 (±2.5)
Area derived diameter (mm)	24.8 (±2.2)
Perimeter derived diameter (mm)	25.4 (±2.3)
Annulus area (mm^2^)	484.7 (±86.0)
Annulus perimeter (mm)	79.9 (±7.0)
Eccentricity index	0.22 (±0.07)
Total calcium-AV (mm^3^)	1680.9 (998.8; 3287.7)
NCC calcium-AV (mm^3^)	628.6 (356.1; 1246.6)
RCC calcium-AV (mm^3^)	573.8 (274.6; 1222.1)
LCC calcium-AV (mm^3^)	532.8 (260; 1014.5)
Total area-AV (mm²)	176.4 (±19.5)
NCC area (mm²)	60.5 (±7.4)
RCC area-AV (mm²)	58.6 (±8.5)
LCC area-AV (mm²)	57.3 (±7.3)
Index total calcium/Total area - AV	9227 (5547; 20,001)
Index NCC calcium/NCC area - AV	9965 (6149; 20,741)
Index RCC calcium/RCC area - AV	9397 (4970; 19,823)
Index LCC calcium/LCC area - AV	8914 (5044; 18,321)

AV, aortic valve; BMI, body mass index; LCC, left coronary cusp; NCC, non-coronary cusp; RCC, right coronary cusp; STS, Society of Thoracic Surgeons.

TAVI implantation took 75.8 (±32.6) minutes with in almost 90% of all patients with need of rapid pacing. Postoperatively, patients stayed for more than 2 days (54.2 (±73.5) hours) on ICU. Overall, 7 patients died. No patient presented severe PVL. Incidence of postoperative mild PVL (grade I) was in 34% of all patients, and of PVL Grade II 3.3% of all patients. In most patients no PVL could be found (62.7%).

### Impact of aortic geometry and calcification degree on PVL

Using a multivariable logistic regression analysis, independent predictors for postoperative incidence of paravalvular leak were calculated (Table 2). Calcium amount of all three cusps together (*p* = .032) as well as of each cusp separately were significant predictors for postoperative incidence of paravalvular leak (Calcium-NCC *p* = .001, Calcium-RCC *p* < .001, Calcium-LCC *p* = .001), whereas the area itself of aortic cusps was not a predictor (*p* > .05).

**Table 2.** Multivariable analysis of MDCT predictors of paravalvular leak.

MDCT measurements	*p*-value
Total calcium-AV	**0.032**
NCC calcium-AV	**0.001**
RCC calcium-AV	**<0.001**
LCC calcium-AV	**0.001**
Total area-AV (mm²)	0.126
NCC area (mm²)	0.214
RCC area-AV (mm²)	0.071
LCC area-AV (mm²)	0.501
Index total calcium/Total area - AV	**0.010**
Index NCC calcium/NCC area - AV	**0.027**
Index RCC calcium/RCC area - AV	**0.007**
Index LCC calcium/LCC area - AV	**0.006**
Annulus area	**0.046**
Annulus perimeter	0.051
Minimum diameter annulus	**0.046**
Maximum diameter annulus	0.076
Area derived diameter	**0.022**
Perimeter derived diameter	**0.038**
Eccentricity index	0.414

AV, aortic valve; LCC, left coronary cusp; NCC, non-coronary cusp; RCC, right coronary cusp.

Not only the Calcium account of each aortic cusp was a predictor, also the index of Calcium amount to the area of aortic valve significantly predicted postoperative incidence of a mild paravalvular leak (Figure 1). Index of NCC-Calcium degree to NCC aortic area was predictive (*p* = .027). Same was calculated for index of total Calcium to total aortic area (*p* = .010) and index of RCC-Calcium to RCC-aortic area (*p* = .007) and index of LCC-Calcium to LCC-aortic area (Table 2). The area of aortic annulus was also a significant predictor (*p* = .046).

**Figure 2.**
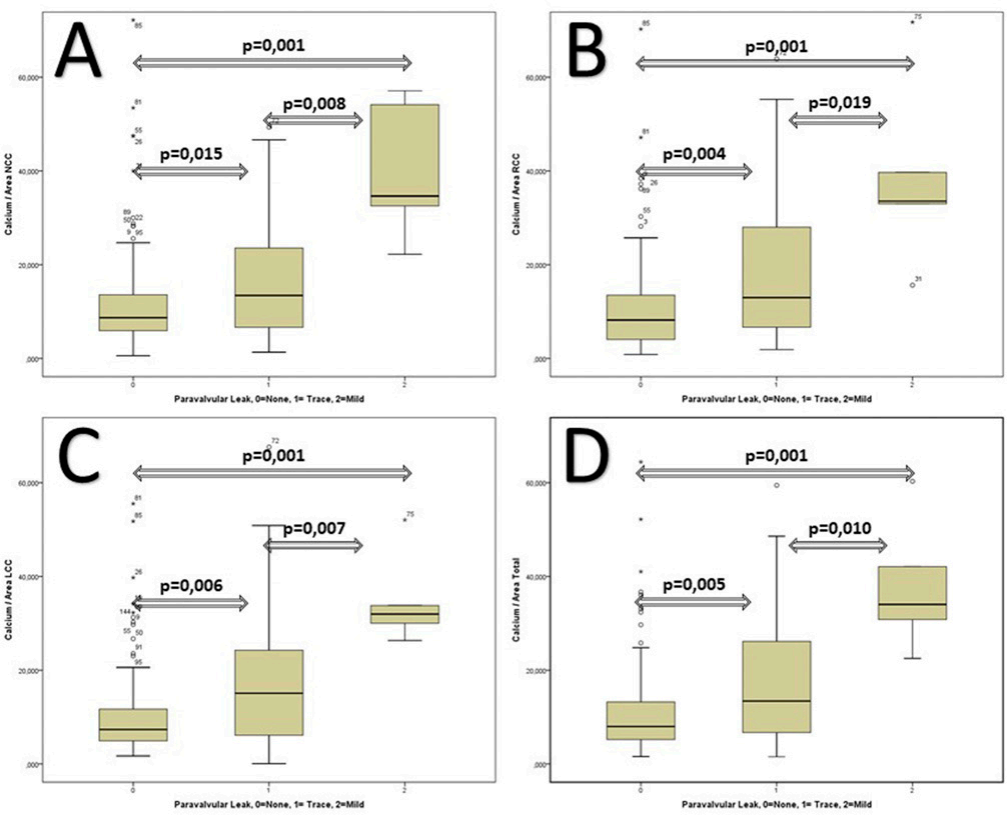
The correlation of the multiple assessments of PVL-Level (0 = None, 1 = Trace and 2 = mild) with different indexed Calcium to Area Values (A = LCC, B = RCC, C = LCC and D = Total).

The Boxplots analysis reveals that there is a strong correlation of indexed Calcium load of the three different coronary cusps to the Area and the postoperative degree of paravalvular leak (Figure 2).

## Discussion

Aortic calcification plays a key role in paravalvular leak occurrence after TAVI procedure.^1,9^ Though continuous advancement in TAVI technology, incidence pf postoperative PVL still remains high.^10^ In well-known PARTNER trial a correlation between the grade of PVL and 1-year mortality was observed.^3^ Therefore, evaluation of preoperative risk factors and optimization of the preoperative diagnostics are necessary to reduce PVL incidence after TAVI procedure. Different methods and strategies for calculation of the calcification degree already exist.^1,5^ However, there is no standardization for optimal calcium quantification.^11,12^ In order to provide best therapy option for each patient a standardized and reproducible method for calcium quantification is necessary. Pollari et al. evaluated impact of calcification degree on incidence of PVL using a similar method as we do.^9^ Jilaihawi et al. presented 2014 a method using contrast and non-contrast CT scans.^5^ This study demonstrates that calcium quantification is fundamentally determined by the individually set HU thresholds to detect calcium without falsification due to contrast agents. But not only the correct calcium quantification is essential also the correlation of calcium degree with surface area of each aortic cusp is relevant. We present a new method considering in contrast-enhanced CT scans different iodine contents for optimized and more accurate calculation and quantification of the calcification degree without falsification due to contrast agents. The aim of our study was to provide a better determination of calcium volume with a more accurate quantitative and reproducible measurement method in order to receive better preoperative information for the planning intervention.

Our diameter of the aortic annulus was similar with the results by Pollari et al. determined by a similar method using CT scan.^9^ In contrast Thourani et al. measured the aortic annulus via 3D echocardiography instead with a CT scan.^13^

Literature revealed different study results in terms of considering contrast and non-contrast CT scans for calcium quantification.^5,14^ Mühlenbruch et al. determined a calcium threshold of 350 HU in contrast-enhanced CT scans and of 130HU in non-contrast CT scans corroborating results of Hong et al.^14,15^ Thereby he found out that choice of calcium threshold and use of contrast agent significantly influenced results of calcium quantification.^14^ However, he did not use an individually set HU threshold for each patient for calcium quantification as we did. Interestingly, in contrast CT scans with calcium threshold of 350HU a 56.2% higher amount of calcium was found. These results may lead to the finding that contrast agents imitate calcium in CT scans leading to false results. Therefore, choice of the adequate calcium threshold is of elementary importance. Finally, Mühlenbruch stated that calcium quantification with contrast-enhanced CT scan is not a reliable method due to falsification of the contrast agent as calcium. Jilaihawi et al. found out that in contrast-enhanced CT scans a calcium threshold of 850HU would be optimal.^5^ This is much higher than the threshold used in our study. Additionally, they did not consider the patient’s individual calcium threshold.

Moreover, Waldschmidt et al. applied a threshold of 550 Hounsfield units to discriminate between calcification and contrast medium in the region of the leaflet and LVOT.^16^ They also report that occasionally this threshold had to be adjusted due to the visual estimation which resulted in a higher mean threshold of 556 HU in their study population. The exact technique was not descripted.

In contrast we used individual calcium thresholds. And not a standard threshold for the complete patient cohort. These thresholds were determined by three voxel probes of non-calcium containing tissue of lowest opacity resulting in a reference value for each individual patient and considering the different iodine content of the contrast agent. Thereby an individual calcium threshold was calculated which was 50HU set above the individual reference value. However, our threshold with 50 HU above the reference value may lead to false quantification of contrast agent as calcium. If these calcium thresholds were high, suggesting that it is only most dense (and therefore bright) calcium that appears to influence PVL and that the discriminatory value for PVL can be enhanced by removing “noise” arising at lower thresholds for detection.

In terms of total amount of calcium Pollari et al. revealed significantly lower results because he chose only two calcium thresholds, whereas we calculated individual thresholds for each patient.^9^ Also, other studies determined an arbitrarily set calcium threshold for all patients.^11,14^ In presence of extensive calcifications and thus increased risk for PVL the procedural strategy should be modified for each patient individually.^9^

Most recently, the presence of even mild residual PVL in high-risk patients was found to be associated with a significantly worse survival compared to patients who did not exhibit such a complication in long-term follow-up.^17,18^ Additionally, in intermediate-risk patients this tendency was also observed for moderate and severe PVL.^9^

It is pivotal to analyse other areas of calcium distribution, such as the annulus, the LVOT, the Sinus of Valsalva or the mitro-aortic continuity. Routinely, the calcium load was measured starting from the LVOT - 3 mm from the annulus to the sinutubular junction. Anees Musallam published in 2022 a paper showing LVOT calcium load predict outcome after TAVR.^19^ In our publication we tried a different approach showing that the calcium load of each cusp is linked to the surface area to each cusp. This highlights our new approach to standardize measurement of calcium load and the need for a new approach to standardize measurement of calcium load taking also into account the surface area of each cusp.^9^”

To the best of our knowledge this is the first clinical method for optimized calculation of the calcification degree considering the individual calcification reference value, individual calcification of each aortic cusp and considering surface area of each aortic cusp. Our results clearly show that our optimized method may be used as an accurate tool to reduce and predict PVL occurrence after TAVI procedure. Moreover, it may help in the decision making and choosing the best prosthesis in a particular situation. Direct comparison of our new method with established methods do not already exist. Further studies are therefore necessary. Despite being more accurate, the main advantage of our newly developed method is that we do not only focus on the aortic calcification degree, we also correlate the calcification degree with the surface area of each aortic cusp. Our method also impresses by its simplicity. It may be calculated instantly either by the surgeon or a resident in almost any clinical situation once the basic parameters have been gathered by the CT diagnostics, imaging and physical examination of the patient at arrival. Computed tomography (as the diagnostic gold standard) is mandatory to allow for information on the extent of calcification degree and aortic root geometry.

## Conclusion

Aortic calcification degree plays a major role in postoperative incidence of PVL after TAVI procedure. Our optimized new method includes a more accurate quantification of the calcification degree of each aortic leaflet and correlates the calcification degree with the surface area of each aortic cusp which enables better determination of postoperative incidence of PV. This may be helpful in the individualized tailored therapy planning.

## Impact on daily practice

Preoperative measurement for TAVI is essential to achieve the best result.

A standardization of the measurement methods leads to a comparability of the studies. Consideration of the anatomical size of each cusp and the respective calcium load can influence the choice of the prosthesis and thus further reduce the complications of the procedure. Our study results need to be evaluated in a larger cohort also analyzing newer valve generations.

## Limitation

The retrospective nature of our study which calls for the need of randomized prospective studies to validate our findings. Until then, suggesting a specific prosthesis for a particular calcification pattern would be imprudent. One of the main study limitations is the small sample analyzed (*n* = 150). Our new method of calcium quantification should be evaluated using news valve type such as Evolut Pro or Navitor. Moreover, besides PVL occurrence also other perioperative complications should not be underestimated.

## Data Availability

The data underlying this article will be shared on reasonable request to the corresponding author.* Presentation of an optimized method for calcium quantification for better PVL prediction.
